# Intraoperative technology in skull base and vascular neurosurgery: a two-stage cross-sectional national survey

**DOI:** 10.1007/s10143-026-04580-8

**Published:** 2026-11-14

**Authors:** Ahmad M. S. Ali, John Hanrahan, Carole Turner, Andrew Alalade, Alexandros Boukas, Dimitrios Paraskevopoulos, Puneet Plaha, Hani J Marcus

**Affiliations:** 1 Department of Neurosurgery, The Walton Centre NHS Foundation Trust, Liverpool, UK; 2 Institute of Systems, Molecular, and Integrative Biology, University of Liverpool, Liverpool, UK; 3 Department of Neurosurgery, University College London Hospitals NHS Foundation Trust, London, UK; 4 UCL Queen Square Institute of Neurology, University College London, London, UK; 5 Division of Neurosurgery, Department of Clinical Neurosciences, University of Cambridge, Addenbrooke’s Hospital, Cambridge, UK; 6 Department of Neurosurgery, Lancashire Teaching Hospitals NHS Foundation Trust, Preston, UK; 7 School of Health, Social Work and Sport, University of Lancashire, Preston, UK; 8 Department of Neurosurgery, Oxford University Hospitals NHS Trust, Oxford, UK; 9 Blizard Institute, Queen Mary University of London, London, UK; 10 Department of Neurosurgery, The Royal London Hospital, Barts Health NHS Trust, London, UK; 11 Department of Neurosurgery, John Radcliffe Hospital, Oxford, UK; 12 Nuffield Department of Clinical Neurosciences, Oxford University, Oxford, UK

**Keywords:** Neurosurgery technology, Intraoperative technology, Surgical innovation, Technology use

## Abstract

Skull base and vascular neurosurgery rely on diverse intraoperative technologies, but contemporary data describing how frequently they are used at the level of individual operations are limited. On behalf of the Society of British Neurological Surgeons (SBNS) MedTech Committee, we aimed to establish a dated descriptive baseline of reported technology use across three operative paradigms. We conducted a two-stage cross-sectional survey of neurosurgeons in the UK and Ireland via the Society of British Neurological Surgeons (SBNS). Six MedTech Committee members compiled a pragmatic technology list. Respondents reported frequency of use for three exemplar procedures: transsphenoidal pituitary adenoma, retrosigmoid vestibular schwannoma, and pterional aneurysm surgery. Use was recorded on a four-point ordinal scale and analysed descriptively, with Kendall’s tau-b used to explore the relationship between experience and reported use. Twenty-four responses from 20 of the 32 adult or mixed adult-paediatric UK units were analysed (11 units per index procedure). Reported use was strongly procedure specific. Neuronavigation was used always or almost always by 8/12 transsphenoidal respondents but infrequently elsewhere. Endoscopy had displaced microscopy in transsphenoidal surgery, whereas microscopy was used always or almost always by all retrosigmoid and pterional respondents. Indocyanine green and micro-Doppler were routine for aneurysms. Neurophysiological monitoring was universal for vestibular schwannoma but evenly divided for aneurysms. Exoscopes, intraoperative MRI and robotics were used infrequently or not at all; augmented reality was reported by 5/13 pterional respondents. No association was found between experience and reported use, though this was underpowered. Reported intraoperative technology use is strongly shaped by operative paradigm, with established technologies clustering around recurring procedural needs. Newer technologies were used infrequently. Because availability was not recorded, non-use cannot be distinguished from lack of access. These data provide a dated baseline against which future change can be assessed.

**Supplementary information:**

The online version contains supplementary material available at https://doi.org/10.1007/s10143-026-04580-8.

## Introduction

Skull base and vascular neurosurgery are among the most technically demanding areas of contemporary neurosurgical practice. These procedures are performed through narrow anatomically complex operative corridors, often around critical neurovascular structures, and depend on accurate approach planning, visualisation, tissue manipulation, and intraoperative monitoring [1].

Modern neurosurgical practice is therefore increasingly supported by intraoperative technologies [2]. These include established adjuncts, such as neuronavigation, endoscopy, microscopy, high-speed drills, ultrasonic aspirators, indocyanine green video angiography, micro-Doppler ultrasonography, and neurophysiological monitoring, as well as newer technologies such as augmented reality, exoscopy, intraoperative MRI, and robotics. However, adoption of these technologies varies according to factors such as operative paradigm, surgeon preference and training, institutional resources, workflow, and perceived clinical value [3].

Despite the rapid development of surgical technology, contemporary national data describing its real-world use in skull base and vascular neurosurgery are limited [4]. Such data serve two purposes. First, they provide a dated baseline against which subsequent change can be measured, which is particularly relevant for technologies currently in active diffusion. Second, they indicate where emerging technologies have, and have not, entered routine use, which is relevant to service planning and to identifying where further evaluation is required.

We therefore conducted a survey of neurosurgeons in the UK and Ireland, on behalf of the MedTech Section of the Academic Committee of the Society of British Neurological Surgeons (SBNS), which commissioned it as a scoping exercise, to characterise reported use of intraoperative technology in skull base and vascular neurosurgery. Specifically, we aimed to describe patterns of reported use across three exemplar operative paradigms: transsphenoidal approaches for pituitary adenomas, retrosigmoid approaches for vestibular schwannomas, and pterional approaches for intracranial aneurysms. The survey was designed to describe current practice rather than to explain it.

## Methods

This was a two-stage cross-sectional national survey developed by the MedTech Section of the Academic Committee of the Society of British Neurological Surgeons (SBNS), which commissioned it as a scoping exercise to establish current practice before advising on technology evaluation and service provision. The study was designed to characterise current use of intraoperative technology in skull base and vascular neurosurgery in the UK and Ireland. Ethical approval was not required: the study surveyed professional practice among clinicians and collected no patient data. Respondent email addresses were recorded by the survey platform to allow duplicate submissions to be identified; they were not used in analysis and are not reported. Distribution was approved by the SBNS.

### Stage 1: selection of technologies

In the first stage, six members of the MedTech Section were asked to identify intraoperative technologies that they were familiar with or had observed in use across neurosurgical centres. For the purposes of this study, intraoperative technologies were defined as adjuncts requiring an external power supply. Responses were reviewed by the committee and grouped into four functional categories: approach planning and navigation, visualisation, tissue manipulation, and intraoperative monitoring. This process was used to generate a predefined list of technologies for inclusion in the national survey. This was a pragmatic, expert-generated list rather than a formal consensus exercise such as a Delphi or nominal group process, and no predefined threshold for inclusion was applied.

### Stage 2: national survey

In the second stage, an online questionnaire was distributed to neurosurgeons in the UK and Ireland through the Society of British Neurological Surgeons mailing list. The survey was hosted on Google Forms and opened on 12 August 2025 and closed on 8 December 2025. It was open to consultants and neurosurgical residents. The questionnaire was circulated to the SBNS membership mailing list. The number of members regularly performing each index procedure was not known, so a procedure-specific response rate could not be calculated.

The survey captured respondent characteristics, including training grade, years of neurosurgical experience and institution of practice. Three index procedures were selected to represent distinct operative paradigms within skull base and vascular neurosurgery - endoscopic transsphenoidal resection of pituitary adenomas, retrosigmoid resection of vestibular schwannomas and pterional clipping of intracranial aneurysms. These procedures were chosen as representative examples of contemporary skull base and vascular practice.

For each exemplar operation, respondents were first asked whether they performed the procedure regularly. Those who did were then asked to report their frequency of use of each predefined intraoperative technology for that procedure. Frequency of use was recorded using a four-point ordinal scale: always or almost always; mostly (> 50% of cases); sometimes (<50% of cases); or never or almost never. Throughout, “routine” use denotes the combined “always or almost always” and “mostly” categories. A four-point scale without a neutral middle option was used to encourage respondents to make a judgement regarding routine use [5]. Respondents could also report additional technologies using a free-text field. All questions were optional.

### Statistical analysis

Responses were analysed descriptively. Categorical variables were summarised using counts and percentages. Each exemplar procedure was analysed independently, including only respondents who reported performing that procedure regularly. Technologies were analysed according to their functional category. Free-text responses were reviewed and grouped thematically. Frequencies are reported across all four response levels with explicit denominators; the full distribution for every technology in every procedure is given in Supplementary Table 1.

For each adjunct, the correlation between experience and reported use was assessed using Kendall’s rank correlation coefficient (tau-b), which accommodates the large number of tied ranks inherent to ordinal data. Adjuncts used identically by all respondents were excluded, and p-values were adjusted within each procedure using the Benjamini–Hochberg procedure. Given the exploratory nature of these analyses and the small sample size, results were interpreted descriptively. Analyses and figures were generated in R version 4.3.1. These analyses were specified as exploratory and are reported in full, including null results.

## Results

### Respondent characteristics

A total of 25 responses were received. One respondent was excluded because they did not report regularly performing any of the exemplar procedures, leaving 24 responses for analysis. Most respondents were consultants (22/24, 92%), and the remainder were neurosurgical residents (2/24, 8%). Respondents were highly experienced with 14/24 (58%) reporting at least 20 years of neurosurgical experience. Responses were received from 20 distinct neurosurgical units in Great Britain, representing 20 of the 32 adult or mixed adult-paediatric neurosurgical units in the UK (63%). No responses were received from units in Northern Ireland or the Republic of Ireland, and none from paediatric-only units (Table 1).

**Table 1:** Summary demographics of survey respondents

		Count (%)
Years of experience	>20 years	14 (58%)
	15-20 years	3 (13%)
	10-15 years	1 (4%)
	5-10 years	5 (21%)
	<5 years	1 (4%)
Location	Scotland	2 (8%)
	North of England	8 (33%)
	South of England	6 (25%)
	London	6 (25%)
	Wales	1 (4%)

Twelve respondents reported regularly performing transsphenoidal approaches for pituitary adenomas, 12 reported regularly performing retrosigmoid approaches for vestibular schwannomas, and 13 reported regularly performing pterional approaches for intracranial aneurysms. Of the 24 respondents, 12 regularly performed one index procedure, 11 performed two, and one performed all three. Eleven distinct units were represented for each of the three procedures.

### Transsphenoidal approaches for pituitary lesions

**Fig. 1:**
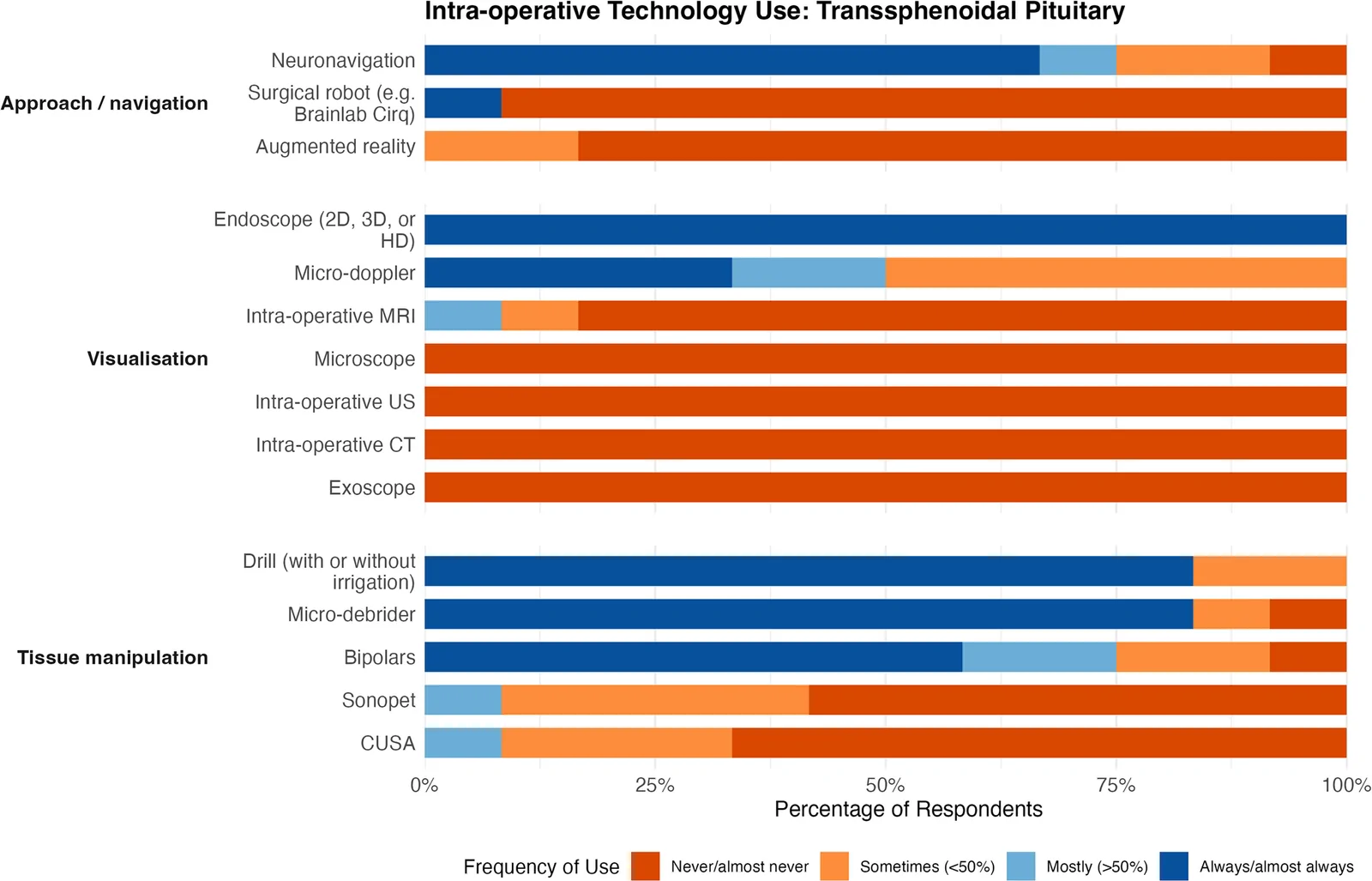
Stacked bar chart demonstrating frequency of adjunct use for transsphenoidal approaches for pituitary lesions organised from most to least used

The frequency of intraoperative technology use for transsphenoidal approaches is shown in Fig. 1. For approach and navigation, neuronavigation was used always or almost always by 8/12 respondents, mostly by 1/12, sometimes by 2/12, and never or almost never by 1/12; it was therefore not part of routine practice for a quarter of respondents. Augmented reality was used sometimes by 2/12, and a surgical robot was used always or almost always by a single respondent.

For visualisation and vascular localisation, endoscopy was universal (all respondents always or almost always), and micro-Doppler ultrasonography was used by every respondent to some degree, although the frequency varied (always or almost always 4/12, mostly 2/12, sometimes 6/12). The microscope, exoscope, intraoperative ultrasound, and intraoperative CT were not used by any respondent, and intraoperative MRI by only a small minority (2/12, 17%).

For tissue manipulation, high-speed drills and micro-debriders were each used always or almost always by 10/12 (83%), and bipolar diathermy was used routinely by most. Ultrasonic aspirators were used by a minority (Sonopet 5/12, CUSA 4/12), mostly sometimes.

In free-text responses, one respondent reported intraoperative cavernous sinus neuromonitoring for extended endonasal cases, and others reported suction monopolar use and Colorado-needle monopolar diathermy.

### Retrosigmoid approaches for vestibular schwannoma

**Fig. 2:**
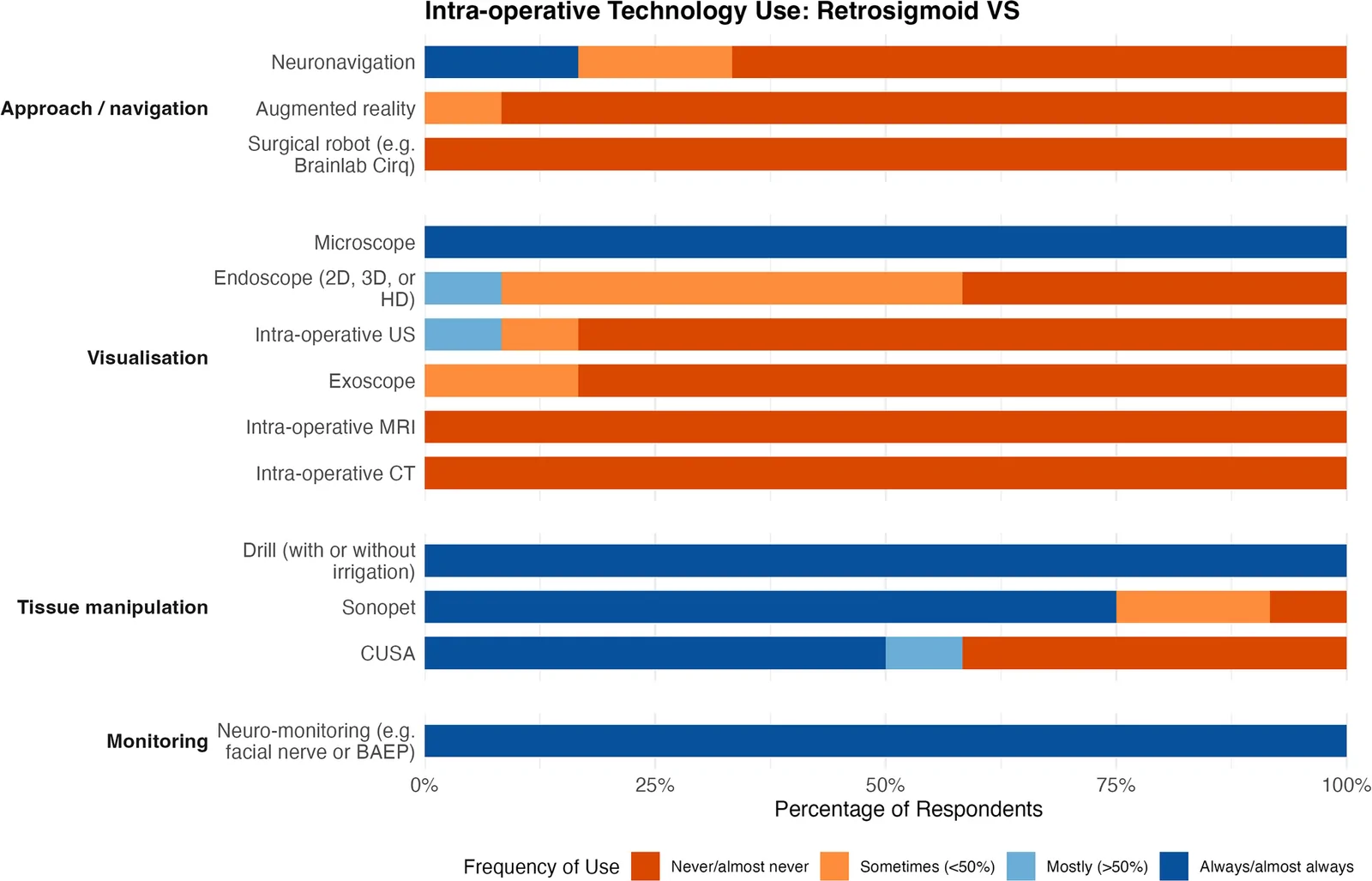
Stacked bar chart demonstrating frequency of adjunct use for retrosigmoid approaches to vestibular schwannomas organised from most to least used

The frequency of intraoperative technology use for retrosigmoid approaches is shown in Fig. 2. For approach and navigation, neuronavigation was used inconsistently (always or almost always 2/12, sometimes 2/12, never or almost never 8/12), augmented reality was used sometimes by one respondent, and surgical robots were not used by any respondent.

For visualisation, the microscope was universal (all respondents always or almost always). Endoscopic assistance was used selectively, with over half of respondents (7/12, 58%) reporting some use. Exoscopy and intraoperative ultrasound were rarely used, with intraoperative MRI and intraoperative CT used by none.

For tissue manipulation, the high-speed drill was universal (all respondents always or almost always), and ultrasonic aspirators were commonly used, with Sonopet reported more frequently (always or almost always 9/12, sometimes 2/12, never 1/12) than CUSA (always or almost always 6/12, mostly 1/12, never 5/12).

For monitoring, intraoperative neurophysiological monitoring (facial nerve or brainstem) was universal, used always or almost always by all respondents.

No additional technologies were identified in free-text responses.

### Pterional approaches for intracranial aneurysms

**Fig. 3:**
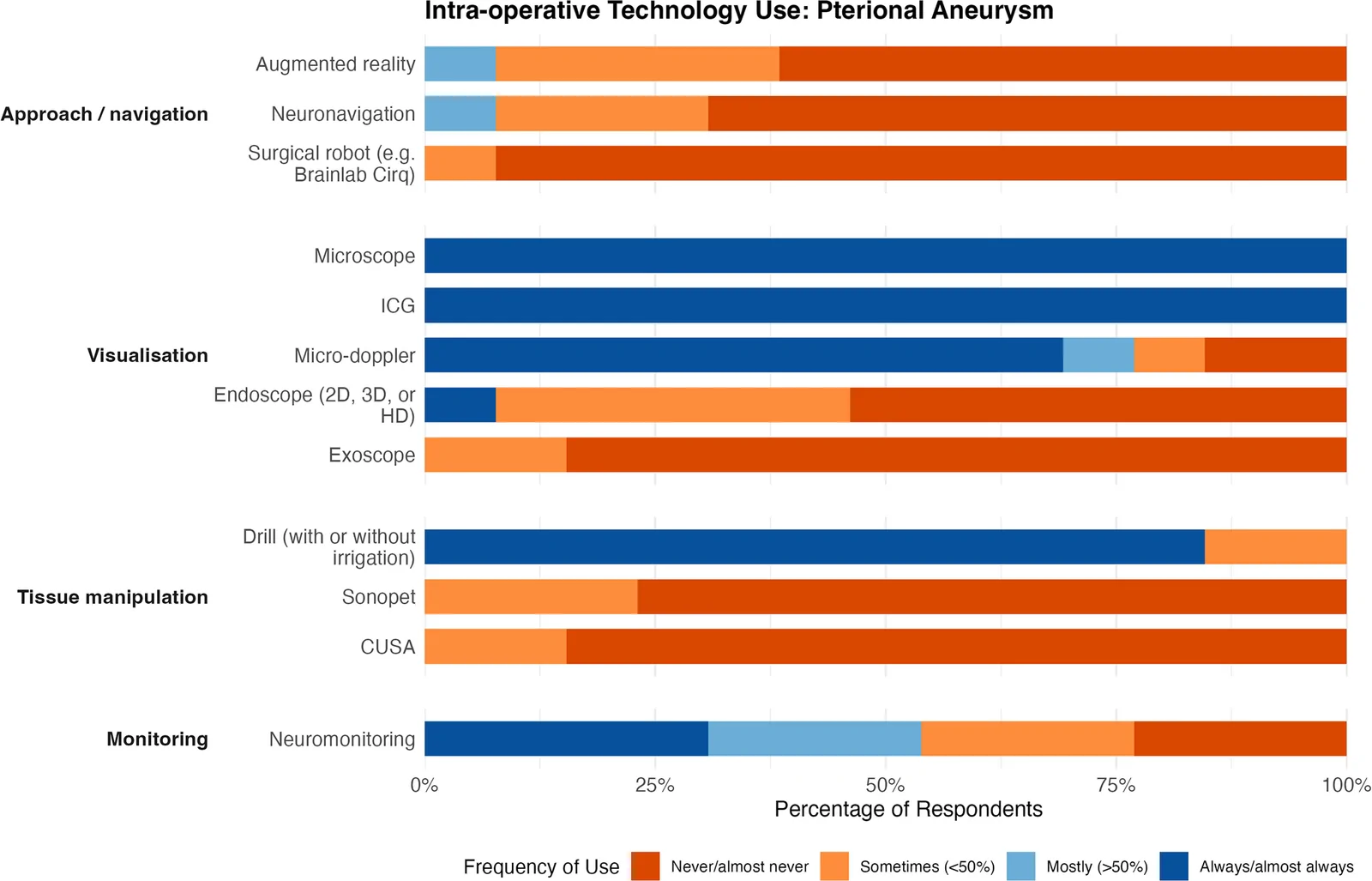
Stacked bar chart demonstrating frequency of adjunct use for pterional approaches for intracranial aneurysms organised from most to least used

The frequency of intraoperative technology use for pterional approaches is shown in Fig. 3. For approach and navigation, neuronavigation was used mostly by 1/13, sometimes by 3/13, and never or almost never by 9/13. Augmented reality was used mostly by one respondent and sometimes by four, giving 5/13 reporting some use, and a surgical robot was used sometimes by one respondent.

For visualisation and vascular localisation, the microscope and indocyanine green video angiography were both universal (all respondents always or almost always). Micro-Doppler ultrasonography was also used commonly, though not universally (routine in 10/13, 77%). Endoscopic assistance was reported by a minority (6/13, 46%), usually as an occasional adjunct and exoscopy was rarely used.

For tissue manipulation, high-speed drills were used always or almost always by most respondents (11/13, 85%), whereas ultrasonic aspirators were used infrequently.

For monitoring, neurophysiological monitoring was divided almost evenly across the scale (always or almost always 4/13, mostly 3/13, sometimes 3/13, never or almost never 3/13).

Free-text responses clarified that the augmented-reality use reported by one respondent was part of a research programme rather than routine clinical practice, and one respondent specified the use of a handheld endoscope.

### Findings diverging from expected practice

Several findings differed from what might be assumed from established practice. Neuronavigation was not used routinely by all transsphenoidal surgeons (always or almost always 8/12). Micro-Doppler ultrasonography was used to some degree by every transsphenoidal respondent. Neurophysiological monitoring in pterional aneurysm surgery was divided evenly across all four response levels. Among ultrasonic aspirators in retrosigmoid surgery, the Sonopet was used always or almost always by 9/12 compared with 6/12 for the CUSA. The exoscope was used sometimes by 2/12 retrosigmoid and 2/13 pterional respondents and by none transsphenoidally, with no evidence of substitution for the operating microscope, which was used always or almost always by every respondent performing those approaches.

### Effect of experience on adjunct use

Surgeon experience showed no statistically significant association with the use of any intra-operative technology in any of the three exemplar procedures. Across all testable technologies, rank correlations were weak and inconsistent in direction (Kendall’s tau-b ranging from − 0.44 to + 0.47), and none reached significance either before or after correction for multiple comparisons (lowest unadjusted *p* = 0.088; lowest FDR-adjusted *p* = 0.49). Several adjuncts were used uniformly by all respondents, including the operating microscope across all three procedures, the drill and facial-nerve or brainstem monitoring in retrosigmoid approaches, and indocyanine green in pterional approaches, and were therefore not amenable to correlation analysis. This analysis was underpowered: with 12 to 13 respondents per procedure and extensive tied ranks, only strong associations could have been detected. The absence of a detected association should therefore be read as uninformative rather than as evidence that no association exists.

## Discussion

### Principal findings

This two-stage cross-sectional national survey provides a contemporary overview of intraoperative technology use in skull base and vascular neurosurgery across Great Britain. Across three exemplar procedures, reported technology use was strongly procedure specific. Each operative paradigm was anchored around a small number of established technologies: endoscopy, neuronavigation, high-speed drills and micro-debriders for transsphenoidal pituitary surgery; microscopy, drilling, ultrasonic aspiration and neurophysiological monitoring for retrosigmoid vestibular schwannoma surgery; and microscopy, indocyanine green video angiography and micro-Doppler ultrasonography for pterional aneurysm surgery.

By contrast, reported use of more recently introduced technologies was infrequent or absent. Augmented reality and the exoscope showed limited, selective use, whereas intraoperative MRI, intraoperative CT and robotic systems were almost entirely absent across all three procedures [6–13]. Surgeon experience was not shown to be associated with frequency of technology use, although the study was not powered to detect such an association [14, 15]. The survey did not measure availability, perceived clinical value, or workflow integration, so the relative contribution of these factors cannot be assessed from these data.

### Results in context

For approach planning and navigation, neuronavigation was most commonly used in transsphenoidal pituitary surgery [16]. This likely reflects the narrow and variable anatomical corridor, the proximity of the internal carotid arteries, and the importance of orientation during endonasal access. In contrast, neuronavigation was used less consistently for retrosigmoid and pterional approaches, where surgeons may rely more heavily on surface anatomy, bony landmarks and microsurgical exposure.

For visualisation, the findings demonstrate a clear division between endoscopic and microscopic operative paradigms. In transsphenoidal surgery, the endoscope had fully displaced the microscope among respondents, consistent with the broader shift towards endoscopic pituitary surgery. In retrosigmoid and pterional approaches, the microscope remained universal, but endoscopic assistance was reported selectively [17–21]. In pterional aneurysm surgery, indocyanine green video angiography was universal and micro-Doppler ultrasonography was commonly used, indicating that vascular visualisation and flow assessment are embedded in routine practice. Both were used routinely by most respondents, although the survey did not record whether they were used in the same cases, and no inference about complementary use can be drawn.

For tissue manipulation, high-speed drilling remained a core technology across all three procedures, although its role differed by approach. In transsphenoidal surgery, drills and micro-debriders were commonly used for nasal and sphenoid work, while ultrasonic aspirators were less frequently reported. In retrosigmoid surgery, drilling was universal and ultrasonic aspirators were commonly used, reflecting the need for controlled bone removal and tumour debulking near critical neurovascular structures. In pterional aneurysm surgery, drills were commonly used, whereas ultrasonic aspirators were rarely required.

For intraoperative monitoring, the pattern was more selective. No routine monitoring technology was reported for transsphenoidal surgery. In retrosigmoid vestibular schwannoma surgery, facial nerve and brainstem monitoring were universal, supporting their status as routine technologies for this procedure [22]. In pterional aneurysm surgery, neurophysiological monitoring showed greater variation, suggesting that its perceived value remains more dependent on surgeon or institutional preference.

These patterns are consistent with the limited comparable literature. A recent national survey of neurosurgical technology use in Germany reported similarly high availability of neuronavigation, neuromonitoring and advanced imaging alongside inconsistent implementation across centres, with more varied technology use reported in academic centres [23]. In another survey of technology use in the USA variable use of ultrasound, microscopes and loupes was found; loupes fell outside our definition of intraoperative technologies as adjuncts requiring an external power supply and were therefore excluded by design [24]. This survey also suggested that trainees’ use of intraoperative equipment tends to mirror that of their supervising surgeons rather than to diverge from it.

The following discussion is offered as a theoretical frame of interest to the reader rather than as a finding of this survey, which recorded what was used and not why. With that caveat, the contrast between frequently and rarely used technologies can be considered through complementary models of innovation diffusion and normalisation. The entrenched adjuncts such as indocyanine green angiography, micro-Doppler ultrasonography, and neurophysiological monitoring, share attributes that favour adoption: a clear relative advantage, straightforward compatibility with existing operative workflow, lower complexity, and high observability, in that they provide immediate, visible intraoperative feedback [25, 26]. Although these factors favour the diffusion of innovations, they do not explain how it becomes embedded. Normalisation Process Theory describes how this occurs through four mechanisms [27]. Normalised technologies cohere with the conceptual aims of the operation, so surgeons share a settled understanding of their purpose (coherence). The surgical community has enrolled around them and legitimated them as components of standard practice (cognitive participation). They are workable within the operative workflow and fit the division of theatre labour without disruption (collective action). Finally, they are no longer consciously appraised as discrete “technologies” but evaluated tacitly as part of how the operation is simply done (reflexive monitoring). This shift from explicit to tacit appraisal is the signature of a normalised practice. The operating microscope, the endoscope, and indocyanine green represent this endpoint in our survey results, with transsphenoidal endoscopy offering a worked example of a technology that appears to have completed this trajectory by fully displacing its predecessor among our respondents. These mappings are illustrative and untested.

Intraoperative MRI and robotic systems, by contrast, were used rarely or not at all. Our data cannot distinguish between three explanations for this: that the technology was not available, that it was available but deliberately not used, or that it was used but confined to selected cases. Diffusion theory would anticipate slow spread for technologies with high complexity, limited trialability, and substantial infrastructure and cost requirements may impede collective and widespread adoption [25]. On that reading they would be better understood as not yet sufficiently diffused than as rejected, although our data cannot establish this [28]. Frameworks for the staged evaluation of surgical innovations such as the IDEAL framework offer a second and equally compatible reading, indicating that the breadth of a technology’s use should be proportionate to the maturity of its evidence base, with limited, selective use by fewer operators being the expected mode at earlier stages of evaluation rather than a marker of under-adoption. On that reading, selective or cautious use where a technology is available might represent evidence-proportionate restraint rather than a failure to adopt [29–31]. Our data are compatible with either reading, and equally with simple unavailability, and cannot adjudicate between them. “Sometimes” on our ordinal scale might indicate targeted deployment or might reflect restricted access; the survey recorded neither indications nor availability.

The variation in reported use may also have implications for equitable access. In a centralised, publicly funded system such as the NHS, capital-intensive technologies may cluster in tertiary and academic centres as seen in the aforementioned German survey [23]. The technologies used universally in our data may by contrast represent core competencies for which training exposure is likely to be important, whereas rarely used technologies cannot readily be trained at scale. This may matter given survey evidence that trainees’ equipment use mirrors that of their supervisors [24].

We did not find clear evidence that surgeon experience was associated with technology use. This finding should be interpreted cautiously, given the limited statistical power of this analysis. Because the analysis was underpowered, it should not be read as evidence against an association between seniority and technology use [14, 15]. Whether established procedural norms, local availability, training pathways, or perceived usefulness are more important determinants of use than seniority remains untested by these data.

### Strengths and limitations

This study has several strengths. It provides data from 20 neurosurgical units, a majority of adult or mixed adult-paediatric units in Great Britain, with 11 units represented for each index procedure. The use of three exemplar procedures allowed comparison across distinct operative paradigms. The four-point ordinal scale distinguished routine from selective use, which surveys recording only availability or binary use cannot do; this distinction accounts for several of the findings reported above, including the non-universal use of neuronavigation in transsphenoidal surgery and the even division in neurophysiological monitoring for aneurysm surgery.

The study also has important limitations. The sample was small: 24 respondents, with 12 to 13 contributing to each procedure and 11 units represented for each. The response rate cannot be calculated because the number of SBNS members regularly performing each index procedure is not known. As with all voluntary surveys the findings are subject to responder bias, particularly towards surgeons with an interest in technology, and responses were self-reported rather than observed. We did not collect individual or unit operative volume, so responses cannot be weighted by caseload. Most importantly, the survey recorded frequency of use but not availability, so deliberate non-use cannot be distinguished from lack of access, and the determinants of use discussed above are inferred rather than measured. The technology list was generated pragmatically by six committee members rather than by a formal consensus method, and may have omitted technologies. Surgical loupes, however, were excluded by design rather than omitted, as they require no external power supply and so fell outside our definition. Several items also lacked granularity: neurophysiological monitoring was recorded as a single item for each procedure. For vestibular schwannoma surgery that item combined facial nerve and brainstem monitoring, and for aneurysm surgery it named no individual modalities, so somatosensory and motor evoked potentials could not be distinguished from one another or from other modalities. No item recorded the indications for selective use. The experience analysis was underpowered, and the survey did not always distinguish routine clinical use from research use. Terms such as augmented reality and surgical robot were not defined in the questionnaire, and responses may reflect differing interpretations of them. Finally, no responses were received from Northern Ireland, the Republic of Ireland, or paediatric-only units.

## Conclusion

These findings provide a dated descriptive baseline of reported practice across a majority of British neurosurgical units, and show that reported intraoperative technology use is strongly shaped by the demands of each operative paradigm. Established technologies appear to cluster around recurring procedural needs: orientation in transsphenoidal surgery, controlled tumour removal and neurophysiological monitoring in retrosigmoid surgery, and vascular visualisation in aneurysm surgery.

Newer technologies, including intraoperative MRI, augmented reality, exoscopy and robotics, were used infrequently or not at all. Whether this reflects limited availability, deliberate restraint proportionate to the current evidence base, or both, cannot be determined from these data [32]. The diffusion and normalisation literatures suggest that trialability, workflow integration, and observable benefit are relevant to how such technologies become established, but this survey was not designed to test that proposition.

Future work should record availability alongside use, capture the indications for selective use, and examine surgeons’ reasoning directly using qualitative methods.

## Supplementary information

Below is the link to the electronic supplementary material.

Supplementary File 1 (DOCX 47.0 KB)

## Data Availability

No datasets were generated or analysed during the current study.
