# Supplementary material for: Intraoperative technology in skull base and vascular neurosurgery: a two-stage cross-sectional national survey
Source: Neurosurg Rev. 2026 Nov 14;49(1):650. doi: 10.1007/s10143-026-04580-8 (PMC13768224; doi:10.1007/s10143-026-04580-8)
Supplement: Supplementary file 1 [file 10143_2026_4580_MOESM1_ESM.docx]

**Supplementary Table 1**

Reported frequency of use of each intraoperative technology, by index procedure. Values are numbers of respondents. Only respondents who reported regularly performing the procedure are included; there were no missing responses. "Routine" use in the main text denotes the combined "always or almost always" and "mostly" categories.

**Endoscopic transsphenoidal resection of pituitary adenoma (n = 12)**

| **Technology** | **Always / almost always** | **Mostly (>50%)** | **Sometimes (<50%)** | **Never / almost never** | **n** |
| --- | --- | --- | --- | --- | --- |
| Neuronavigation | 8 | 1 | 2 | 1 | 12 |
| Bipolars | 7 | 2 | 2 | 1 | 12 |
| Exoscope | 0 | 0 | 0 | 12 | 12 |
| Microscope | 0 | 0 | 0 | 12 | 12 |
| Endoscope (2D, 3D, or HD) | 12 | 0 | 0 | 0 | 12 |
| Drill (with or without irrigation) | 10 | 0 | 2 | 0 | 12 |
| CUSA | 0 | 1 | 3 | 8 | 12 |
| Sonopet | 0 | 1 | 4 | 7 | 12 |
| Micro-doppler | 4 | 2 | 6 | 0 | 12 |
| Micro-debrider | 10 | 0 | 1 | 1 | 12 |
| Intra-operative MRI | 0 | 1 | 1 | 10 | 12 |
| Intra-operative CT | 0 | 0 | 0 | 12 | 12 |
| Intra-operative US | 0 | 0 | 0 | 12 | 12 |
| Augmented reality | 0 | 0 | 2 | 10 | 12 |
| Surgical robot (e.g. Brainlab Cirq) | 1 | 0 | 0 | 11 | 12 |

**Retrosigmoid resection of vestibular schwannoma (n = 12)**

| **Technology** | **Always / almost always** | **Mostly (>50%)** | **Sometimes (<50%)** | **Never / almost never** | **n** |
| --- | --- | --- | --- | --- | --- |
| Neuronavigation | 2 | 0 | 2 | 8 | 12 |
| Exoscope | 0 | 0 | 2 | 10 | 12 |
| Microscope | 12 | 0 | 0 | 0 | 12 |
| Endoscope (2D, 3D, or HD) | 0 | 1 | 6 | 5 | 12 |
| Drill (with or without irrigation) | 12 | 0 | 0 | 0 | 12 |
| CUSA | 6 | 1 | 0 | 5 | 12 |
| Sonopet | 9 | 0 | 2 | 1 | 12 |
| Neuro-monitoring (e.g. facial nerve or BAEP) | 12 | 0 | 0 | 0 | 12 |
| Augmented reality | 0 | 0 | 1 | 11 | 12 |
| Surgical robot (e.g. Brainlab Cirq) | 0 | 0 | 0 | 12 | 12 |
| Intra-operative CT | 0 | 0 | 0 | 12 | 12 |
| Intra-operative MRI | 0 | 0 | 0 | 12 | 12 |
| Intra-operative US | 0 | 1 | 1 | 10 | 12 |

**Pterional clipping of intracranial aneurysm (n = 13)**

| **Technology** | **Always / almost always** | **Mostly (>50%)** | **Sometimes (<50%)** | **Never / almost never** | **n** |
| --- | --- | --- | --- | --- | --- |
| Neuronavigation | 0 | 1 | 3 | 9 | 13 |
| Exoscope | 0 | 0 | 2 | 11 | 13 |
| Microscope | 13 | 0 | 0 | 0 | 13 |
| Endoscope (2D, 3D, or HD) | 1 | 0 | 5 | 7 | 13 |
| Drill (with or without irrigation) | 11 | 0 | 2 | 0 | 13 |
| Micro-doppler | 9 | 1 | 1 | 2 | 13 |
| ICG | 13 | 0 | 0 | 0 | 13 |
| CUSA | 0 | 0 | 2 | 11 | 13 |
| Sonopet | 0 | 0 | 3 | 10 | 13 |
| Neuromonitoring | 4 | 3 | 3 | 3 | 13 |
| Augmented reality | 0 | 1 | 4 | 8 | 13 |
| Surgical robot (e.g. Brainlab Cirq) | 0 | 0 | 1 | 12 | 13 |
